# Comparison of Pulse Wave Arrival Times Measured by Bioimpedance Method and Doppler Ultrasound

**DOI:** 10.33549/physiolres.935759

**Published:** 2025-12-01

**Authors:** Eva ZAVODNA, Ladislav SOUKUP, Ivo VIŠČOR, Pavel JURÁK, Jana HRUŠKOVÁ, David KAMPO, Vlastimil VONDRA

**Affiliations:** 1 Department of Physiology and Pathophysiology, Faculty of Medicine, University of Ostrava, Ostrava, Czech Republic; 2 Department of Psychology, Faculty of Arts, Masaryk University, Brno, Czech Republic; 3 International Clinical Research Center, St. Anne’s University Hospital Brno, Brno, Czech Republic; 4 Institute of Scientific Instruments of the Czech Academy of Sciences, Brno, Czech Republic; 5 Department of Internal Medicine, Hospital of the Merciful Brothers, Brno, Czech Republic

**Keywords:** Pulse wave arrival time, Pulse wave velocity, Pulse wave Doppler, Blood flow, Bioimpedance

## Abstract

Pulse wave velocity is a key indicator of vascular health. This study compared pulse wave arrival time (PWAT) measurements obtained by non-invasive bioimpedance with Doppler ultrasound, the gold standard, at multiple arterial sites. Simultaneous PWAT measurements were performed using bioimpedance (−dZ_MAX_) and Doppler ultrasound (t_1_ – the beginning of the blood flow, t_2_ – the peak of the blood flow, t_3_ – the end of blood flow, t_1–2_ – the midpoint of the blood flow/center between t_1_ and t_2_) in healthy volunteers. The agreement between R-dZ_MAX_ and the Doppler-derived time points was assessed at various locations. The lowest average error (2.46 %) was observed between R-dZ_MAX_ and t_1–2_, the increase at the midpoint of the systolic flow. However, significant discrepancies were found when comparing R-dZ_MAX_ with t_1_, t_2_, and t_3_. The level of agreement also varied according to the arterial site. Bioimpedance shows promise for PWAT estimation, particularly when R-dZ_MAX_ is used to estimate t_1–2_ derived from Doppler ultrasound, representing the phase of maximal systolic flow acceleration. This suggests that the maximum rate of impedance change during rapid arterial filling provides a more accurate PWAT measurement. More research is warranted to refine bioimpedance techniques for a reliable vascular assessment under various conditions.

## Introduction

Pulse wave velocity (PWV) has evolved over the past two decades from a research parameter to a widely accepted clinical tool for assessing arterial stiffness and cardiovascular risk. As a non-invasive marker directly related to arterial compliance, PWV plays a central role in the evaluation of patients with hypertension [1,2], diabetes [3,4], obesity [5,6], and other cardiovascular risk factors [7–9]. Recent studies have highlighted its prognostic value, demonstrating that an increase of 1 m/s in aortic PWV is correlated with an increase in cardiovascular mortality [10].

Despite its growing clinical relevance, the methodology of PWV measurement remains a subject of debate. Numerous commercial devices attempt to estimate the PWV of the aortic using peripheral measurement sites, such as carotid-femoral, carotid-radial [11], or ankle-brachial indices [12,13], followed by algorithmic conversions to derive central values [11]. Although systems such as cuff-based devices [12,13] offer convenience and ease of use, and applanation tonometry [11] provides greater accuracy. The cuff-based methods of PWV estimation use pressure cuffs at the four extremities in combination with cardiac microphone to calculate overall time of the cardiac-ancle time propagation. The applanation tonometry is based on measuring the distance between two superficial arteries and the time it takes for the pulse wave measured by the mechanical contact method to carry that distance. Both mentioned methods face limitations in accessibility, cost, and operator skill requirements. High-precision Doppler ultrasound methods [14], although capable of directly measuring PWV, are generally too expensive and complex for routine or point-of-care applications.

Furthermore, for certain populations of patients, such as people with diabetes mellitus and peripheral vascular complications, a method capable of segmental PWV in the lower extremities is especially valuable. However, few techniques allow such targeted measurements while remaining affordable and easy to operate by mid-level medical personnel.

The bioimpedance method emerges as a promising alternative offering a simple and low-cost approach for segmental PWV measurement. By detecting changes in electrical impedance associated with the arrival of pulse waves, bioimpedance systems can localize specific arterial segments using surface electrodes, without the need for advanced imaging or highly trained operators.

In this study, conducted within the framework of the Knowledge Transfer to Business (KTB) project FW01010543 (details see in the acknowledgements), our objective was to validate the accuracy of the bioimpedance method for detecting pulse wave arrival times (PWAT) by comparing it directly with the pulsed wave (PW) Doppler ultrasound technique. PWAT involves events ranging from electrical activation of the ventricles, ejection of the ventricles creating a pulse wave in the aortic root, and subsequent transit to the measured site on the artery. This last part is then an important time parameter for PWV calculation. Specifically, we investigated the influence of varying external thigh pressures on arrival times in the carotid and femoral arteries.

## Methods

### Subjects

A total of 35 healthy volunteers, 18 men and 17 women aged 18 to 66 years, participated in this study. The anthropometric parameters are shown in Table 1. None of the volunteers had hypertension, cardiovascular disease or diabetes mellitus or was taking any regular medication at the time of the study. The volunteers were examined in a supine position. All of them were fully conscious and cooperative during the examination.

The examinations of the volunteers were carried out in the Flexible Laboratories of Biomedical Engineering (BME) at St. Ann’s University Hospital in Brno. The Hospital Ethics Committee approved the study and all participants gave their informed consent.

### Data acquisition

We examined six pairs of sites (on the right and left sides) in the neck (a. carotis), upper (a. brachialis, a. radialis) and lower extremities (a. femoralis, a. poplitea, a. tibialis anterior). At each site, bioimpe-dance and Doppler ultrasound were recorded simultaneously. Synchronization of both devices (bioimpedance device and ultrasound) was performed using an external rectangle signal generator, which sent a 5 ms pulse to the Multichannel Bioimpedance Monitor (MBM) analogue input and to the external AUX input of the ultrasound device at the same time. Subsequently, the same beats could later be identified in ultrasound, ECG and bioimpedance during data processing.

#### Bioimpedance measurement

Bioimpedance data were acquired with the Multichannel Bioimpedance Monitor (MBM) [15] that could simultaneously measure the bioimpedance signal up to 18 channels. MBM digitized the ECG signals with bioimpedance signals synchronously.

##### Current electrodes

**Fig. 1:**
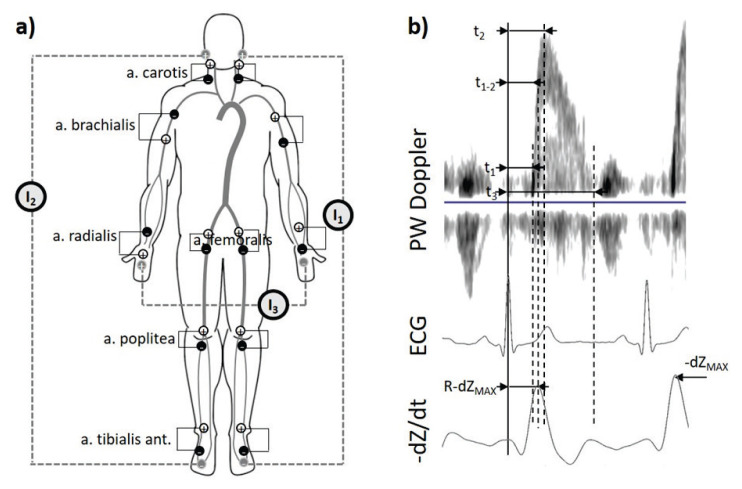
(**a**) Placement of voltage electrode pairs (white – positive; black-negative) for individual measurement sites over arteries, and placement of current electrodes (gray). (**b**) demonstration of determination of particular parameters for arrival times (t_1_, t_2_, t_3_, t_1–2_,R-dZ_MAX_) derived from PW Doppler, ECG and bioimpedance signal −dZ/dt.

Due to the large amount of channel, three different current sources (G) were used for information separation among different parts of the body: (1) G1 (f_1_=49 kHz) between the current electrodes placed behind a left ear and the dorsum of the left leg for the measurement sites in the neck and lower extremities of the left site of the body; (2) G2 (f_2_=50 kHz) between the current electrodes placed behind a right ear and the dorsum of the right leg for the measurement sites in the neck and lower extremities of the right site of the body; (3) (f_3_=51 kHz) between the current electrodes placed on the dorsum of the left and right hand for the measurement sites in the upper extremities (Fig. 1a).

##### Voltage electrodes

A pair of positive (+) and negative (−) voltage electrodes was placed around each measurement point. The description of the exact placement is seen in Fig. 1a:

- A. Carotis:
  (+) 2 cm below the auricle left/right
  (−) Left/right supraclavicular fossa
- A. Brachialis:
  (+) The anterior part of the left upper arm, under the shoulder/ the medial part of the right arm above the a. brachialis.
  (−) The medial part of the left arm above the a brachialis/the anterior part of the right upper arm, under the shoulder.
- A. Radialis:
  (+) The volar part of the right hand just above the wrist/ the volar side of the left forearm 4 cm from the wrist.
  (−) The volar side of the right forearm 4 cm from the wrist/the volar part of the left hand just above the wrist.
- A. Femoralis:
  (+) Inguinal region (torso) 2 cm above the palpable artery left/right.
  (−) The inguinal region (thigh) 2 cm below the palpable artery (shin) left/right.
- A. Poplitea:
  (+) The dorsal part of knee 2 cm above the knee (lower part of the thigh) left/right.
  (−) The dorsal part of the knee 2 cm below the knee (upper part of the thigh) left/right.
- A. Tibialis:
  (+) The medial part of the ankle (shin) left/right.
  (−) The dorsal part of the leg near the ankle left/right.

12 lead ECG signals (ISI BRNO ECG12, The Institute of Scientific Instruments, Brno, Czech Republic) were measured simultaneously with bioimpedance signals. The ECG signals of the ECG were sampled at 500 Hz.

#### Pulse wave ultrasound measurement

We have used a VividTM E9, GE Healthcare, with an M5S-D transducer in PW Doppler mode. The PW Doppler flow was obtained from the six places on the neck, upper, and lower extremities. The linear field probe was placed over the artery of interest, centered between a pair of electrodes (+/−) (Fig. 1a) measuring the bioimpedance signal. After finding the best image position, the Doppler PW recording was started for about 30 s. The accredited clinical biomedical engineer performed the ultrasound measurement.

#### Data processing

The heartbeats of the monitored sites were found to correspond to the processing of the bioimpedance signal, ECG and PW Doppler recordings. This was made possible by recording the synchronizing pulse simultaneously in MBM, and the ultrasound in the separate acquisition channels. The three consecutive cardiac cycles were then visually identified. These were those in which both recordings (bioimpedance and PW Doppler) were all recordings have less noise and artefacts simultaneously.

Bioimpedance signals (Fig. 1b) were filtered by bandpass (Butterworth IIR filter; 0.6–18 Hz), then the signals were derived and inverted. The pulse wave was automatically detected at the maximum value as the –dZ/dt_MAX_ (Fig. 1b) for each channel measured channel. The pulse wave arrival time (R-dZ_MAX_) for the bioimpedance channel was determined as the time from the ECG R-wave to the maximal value of −dZ/dt bioimpedance signal. Signal processing was performed using Matlab.

The duration of the cardiac cycle were automatically determined between two R-R waves of the lead II ECG. In the PW Doppler flow recording, the following pulse wave arrival time parameters were manually determined, based on prior experience [16], for each of the chosen beat: (1) t_1_ – time from R-wave in the ECG to the front foot of the flow curve of PW Doppler; (2) t_2_ – time from the R wave in the ECG to the peak velocity of the main blood flow of PW Doppler; (3) t_1–2_ – calculated as the arithmetic mean of t_1_ and t_2_ for every beat; (4) t_3_ – time from the R-wave in the ECG to the back foot of the flow curve of PW Doppler (Fig. 1b). EchoPAC PC software (GE Healthcare) was used; version 113, was used.

#### Statistical analysis

The Statistica software TIBCO Statistica® 14.0.0.15 was used for statistical analysis. The normal distribution of the observed data was rejected by the Shapiro-Wilk test, therefore, nonparametric tests were used for the following analysis: Spearman’s correlation analysis was used for the correlation, and Wilcoxon’s matched pair test was used to detect the difference between groups. Due to the large number of comparisons, the Holm-Bonferroni correction method was subsequently used. To verify which Doppler-derived arrival time is closest to the bioimpedance-derived parameter, we calculated the percentage error ((t_x_ - R-dZ_MAX_)/t_x_) when particular Doppler-derived arrival times were taken as the true values.

## Results

The raw data of all 35 volunteers were of a high enough quality to allow further analysis. To clarify the relationship between the bioimpedance parameter R-dZ_MAX_ and the time-related parameters obtained from the Doppler ultrasound recordings, we used the nonparametric Spearman correlation. From the results (Table 2), it can be seen that of the parameters, t_3_ correlates least with R-dZ_MAX_, on the other hand, t_1_ and t_1–2_ had the best correlation with R-dZ_MAX_. When looking at individual body parts, the parameters measured on the neck generally show the worst correlation, and the parameters measured on the legs show the best correlation.

**Fig. 2:**
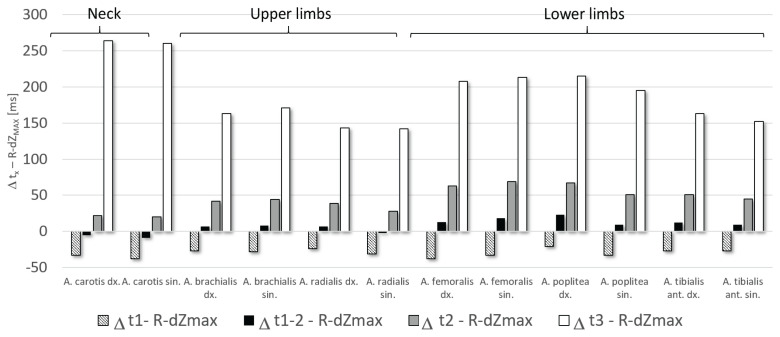
Time differences between R-dZ_MAX_ and particular PW Doppler derived arrival times.

The mean values and standard deviations of the pulse wave arrival time parameters are shown in Table 3. The largest significant differences were found between R-dZ_MAX_ and t3, and also highly significant differences were found between R-dZ_MAX_ and t_1_ and t_2_. On the contrary, the lowest difference was found between R-dZ_MAX_ and the parameter t_1–2_ (Fig. 2).

Table 4 shows the percentage errors between the R-dZ_MAX_ and the arrival times derived from the PW Doppler ultrasound for the different measured sites. The lowest average error (2.46 %) is between R-dZ_MAX_ and the arrival time t_1–2_, which is approximately halfway through the time of increase in blood flow velocity determined by Doppler ultrasound. On average, R-dZ_MAX_ differs by −20.2 % from the t_1_ parameter and by 17.8 % from the t_2_ parameter. The relationship between R-dZ_MAX_ and t_3_ shows the highest error – 47.9 %.

## Discussion

The present study aimed to verify the accuracy of pulse wave arrival time (PWAT) measurements obtained using bioimpedance in comparison to Doppler ultrasound at various arterial locations (carotid, upper limb, and lower limb). Our findings reveal a nuanced relationship between these two methodologies, with the level of agreement varying depending on the specific parameter extracted from the bioimpedance signal and the arterial site under investigation.

A significant finding was the overall average error (2.46 %) found when comparing the R-dZ_MAX_ point on the bioimpedance signal with the arrival time t_1–2_ derived from Pulse Wave Doppler ultrasound. The t_1–2_ time point, crucially, corresponds to the period encompassing the most rapid increase in blood flow velocity during systole – approximately halfway through the systolic upstroke. This strong correlation suggests that the R-dZ_MAX_ point in the bioimpedance signal is a particularly reliable marker for estimating this specific, dynamically active phase of the pulse-wave arrival. We propose that the heightened precision observed with the propose that the heightened precision observed with the t_1–2_ interval is directly related to the underlying hemodynamics of the cardiac cycle. During the early systolic ejection phase, the pulse wave propagates rapidly through the arterial tree, leading to a maximal rate of arterial expansion as the arteries fill with blood. This rapid volume influx and subsequent arterial distension cause the most significant and rapid changes in tissue electrical conductivity, which is precisely what bioimpedance measures [17,18]. Consequently, the rate of change of impedance (−dZ/dt) reaches its maximum (−dZ/dt_MAX_) during this period of maximal arterial expansion driven by the maximal increase in pulse wave velocity. The clear and pronounced change in electrical signal associated with this maximal hemodynamic activity likely contributes to a more accurate and less noise-susceptible detection of pulse wave arrival time by bioimpedance at the R-dZ_MAX_ point.

On the contrary, comparisons of R-dZ_MAX_ with other Doppler-derived time points (t_1_, t_2_ and t_3_) revealed substantial discrepancies. These differences underscore the complexity of directly equating singular points on the bioimpedance waveform with potentially less dynamic or differently influenced phases of the Doppler flow profile. The bioimpedance signal, reflecting volume changes and tissue conductivity [19], may not linearly track the entirety of the blood flow velocity waveform in a simple point-to-point manner.

Variation in agreement across different arterial sites further highlights the influence of pulse wave propagation and properties of the arterial wall. As the pulse wave travels distally, its morphology changes due to wave reflections and variable arterial stiffness, which can affect the timing and magnitude of changes in both blood flow velocity and the associated impedance variations in different ways [20].

Our findings align with existing literature that acknowledges the challenges in directly comparing different PWV and PWAT measurement techniques. For example, Aria *et al*. have investigated pulse wave velocity using bioimpedance in different age groups [21] and compared noninvasive pulse transit time determined from Doppler aortic flow and multichannel bioimpedance plethysmography [16]. While bioimpedance offers advantages in terms of ease of use and cost-effectiveness, our study emphasizes the importance of focusing on specific bioimpedance-derived parameters, such as R-dZ_MAX_ in relation to the t_1–2_ interval, to achieve optimal accuracy in PWAT estimation. The strong correlation observed during the phase of maximal pulse wave velocity increase and corresponding maximal arterial expansion provides a compelling rationale for the precision of PWAT measurements during this specific time window. Doppler ultrasound itself is considered a reliable method for assessing pulse wave velocity and arterial stiffness and is often used as a reference method [22].

Future research should further explore the physiological underpinnings of this relationship, potentially using advanced imaging techniques to simultaneously visualize arterial diameter changes and measure blood flow velocity and impedance variations in real-time. Refining the algorithms for PWAT extraction from bioimpedance signals, particularly focusing on the detection of R-dZ_MAX_ in relation to the maximal systolic flow acceleration, could significantly enhance the clinical utility of this non-invasive technique for vascular assessment. Standardization of measurement protocols and the development of calibration equations could improve the accuracy and clinical applicability of bioimpedance for vascular assessment.

Despite the promising agreement observed for the R-dZ_MAX_ and t_1–2_ relationship, a critical assessment of the inherent methodological limitations of the bioimpedance technique is necessary to fully contextualize the variability observed, especially when comparing against less dynamic Doppler-derived points.

The primary challenge lies in the nature of the bioimpedance signal, which reflects changes in tissue electrical conductivity (volumetric pulsation) and can thus be more susceptible to external and physiological influences not shared by Doppler ultrasound. Key limitations include electrode placement and motion artifacts: The accurate and reproducible positioning of electrodes is crucial. Even minimal electrode displacement [23] or patient movement during the measurement process can significantly alter the baseline impedance and distort the derived signal (dZ/dt), compromising the reliable detection of R-dZ_MAX_. Although impedance measures the entire volume between the electrodes integrally, when determining the velocity of propagation of the pulse wave, localization is ensured by placing the electrodes very close to each other – reducing the volume to the smallest possible part, which will probably eliminate the error from incorrect electrode placement commonly observed in classical body composition. We propose that placing the electrodes in close proximity could reduce the influence of electrode misplacement errors commonly seen in standard bioimpedance setups, although this assumption is currently based on theoretical reasoning and remains to be experimentally confirmed.

Influence of tissue composition: Impedance is strongly modulated by the amount and distribution of blood within the arteries. Fat, muscle mass and bones within the measured segment are not so important for our measurement due to its unchanging nature.

The substantial discrepancies observed when comparing R-dZ_MAX_ to other Doppler-derived points (t_1_, t_2_, t_3_) necessitate a discussion on the inherent physiological and methodological time shift (offset) between bioimpedance and flow-based parameters.

Although bioimpedance reflects the arrival and subsequent distension of the arterial wall (a pressure-wave related phenomenon), and Doppler measures blood flow velocity (a flow-wave related phenomenon), these two signals are not perfectly in phase. Physiologically, the pressure wave (which causes arterial distension) is expected to arrive at a given site slightly earlier than the peak or maximal acceleration of the flow wave due to the inertia of the blood volume. Our finding of a strong correlation between R-dZ_MAX_ and t_1–2_ suggests that both parameters reliably capture the moment of maximal systolic acceleration and subsequent maximal volume influx.

Motion artifacts: A significant element of recording interference are motion artifacts, which can completely prevent the evaluation of measurements. However, all of the above measurement methods, including ultrasound, are affected by this.

## Conclusions

This study confirms that bioimpedance-derived pulse wave arrival times, particularly R-dZ_MAX_, are strongly correlated with early Doppler ultrasound parameters, notably t_1_ and t_1–2_, across multiple arterial sites. The minimal percentage error and strong statistical correlation indicate that bioimpedance is a valid and reliable alternative for assessing pulse wave velocity. Given its simplicity, affordability, and high correlation with established methods, the bioimpedance approach represents a promising tool for non-invasive vascular assessment, with potential for broad clinical implementation in both preventive care and disease monitoring.

The use of bioimpedance for clinical use is much simpler and more accurate than ultrasound-based methods (*bioimpedance allows simultaneous measurement of two sites*), and therefore there is potential to expand bioimpedance into such clinical applications as diabetology and other fields focusing on arterial diseases.

## Figures and Tables

**Table 1:** Anthropometric parameters of the volunteers.

	Age [years]	Weight [kg]	Height [cm]	BMI [kg/m^2^]	Arm span [cm]
*Men*	25.7 (21.6–29.0)	74.9 (67.0–90.0)	179.5 (177.0–183.0)	22.4 (20.7–27.9)	181.5 (177.0–186.0)
*Women*	25.6 (23.2–37.6)	66.8 (59.2–73.3)	167.8 (164.6–173.5)	22.8 (20.8–25.4)	167.0 (163.0–172.5)
*All*	25.7 (22.8–29.0)	70.8 (62.0–83.5)	176.2 (167.5–180.0)	22.5 (20.7–27.7)	176.5 (169.0–183.0)

Median (Q25%–Q75%).

**Table 2:** Spearman correlation coefficients and significances between arrival times measured from bioimpedance (R-dZ_MAX_) and Doppler ultrasound time arriving parameters.

*Artery*	R-dZ_MAX_ vs. t_1_	R-dZ_MAX_ vs. t_1–2_	R-dZ_MAX_ vs. t_2_	R-dZ_MAX_ vs. t_3_
*A. carotis dx*.	0.47***	0.37***	0.27**	0.20
*A. carotis sin*.	0.69***	0.61***	0.45***	0.16
*A. brachialis dx*.	0.38***	0.37***	0.33**	−0.01
*A. brachialis sin*.	0.72***	0.69***	0.65***	−0.04
*A. radialis dx*.	0.87***	0.84***	0.79***	0.09
*A. radialis sin*.	0.80***	0.78***	0.70***	0.11
*A. femoralis dx*.	0.80***	0.80***	0.74***	0.20
*A. femoralis sin*.	0.81***	0.84***	0.83***	0.28*
*A. poplitea dx*.	0.73***	0.71***	0.60***	0.03
*A. poplitea sin*.	0.87***	0.89***	0.85***	0.55***
*A. tibialis ant. dx*.	0.83***	0.88***	0.87***	0.38***
*A. tibialis ant. sin*.	0.76***	0.73***	0.67***	0.33**

R-dZ_MAX_: the time from the ECG R-wave to the maximal value of −dZ/dt bioimpedance signal; t_1_: time from R-wave to the front foot of the flow curve of PW Doppler; t_2_: time from the R wave to the peak velocity of the main blood flow of PW Doppler; t_1–2_: the arithmetic mean of t_1_ and t_2_; t_3_: time from the R wave to the back foot of the flow curve of PW Doppler; statistical significance:

* p<0.05,

** p<0.01,

*** p<0.001.

**Table 3:** The mean values and standard deviations of the particular pulse wave arrival time parameters with the differences detected by the Wilcoxon’s matched pair test between the R-dZ_MAX_ on one side, and parameters derived from PW Doppler (t_1_, t_2_, t_3_, t_1–2_) on the second side.

*Artery*	R-dZ_MAX_ [ms]	t_1_ [ms]	t_1–2_ [ms]	t_2_ [ms]	t_3_ [ms]
*A. carotis dx*.	131 ± 23	100 ± 14***	126 ± 19	153 ± 29**	394 ± 39***
*A. carotis sin*.	136 ± 21	96 ± 15***	127 ± 19*	157 ± 28***	394 ± 28***
*A. brachialis dx*.	165 ± 32	137 ± 20***	172 ± 22*	206 ± 26***	325 ± 59***
*A. brachialis sin*.	164 ± 23	135 ± 22***	171 ± 24*	208 ± 28***	332 ± 60***
*A. radialis dx*.	186 ± 21	162 ± 21***	193 ± 24*	225 ± 28***	327 ± 60***
*A. radialis sin*.	193 ± 23	162 ± 21***	191 ± 22	221 ± 25***	335 ± 66***
*A. femoralis dx*.	218 ± 34	179 ± 30***	230 ± 35**	280 ± 42***	423 ± 50***
*A. femoralis sin*.	215 ± 36	182 ± 33***	233 ± 34***	284 ± 37***	425 ± 38***
*A. poplitea dx*.	229 ± 44	209 ± 36**	252 ± 35*	296 ± 36**	443 ± 30**
*A. poplitea sin*.	235 ± 41	202 ± 31**	244 ± 32	286 ± 34**	430 ± 32**
*A. tibialis ant. dx*.	274 ± 35	248 ± 34***	287 ± 33*	326 ± 35***	438 ± 33***
*A. tibialis ant. sin*.	273 ± 36	244 ± 37***	281 ± 39*	317 ± 41***	423 ± 45***

R-dZ_MAX_: the time from the ECG R-wave to the maximal value of −dZ/dt bioimpedance signal; t_1_: time from R-wave to the front foot of the flow curve of PW Doppler; t_2_: time from the R wave to the peak velocity of the main blood flow of PW Doppler; t_1–2_: the arithmetic mean of t_1_ and t_2_; t_3_: time from the R wave to the back foot of the flow curve of PW Doppler; statistical significance:

* p<0.05,

** p<0.01,

*** p<0.001.

**Table 4:** Error estimation in percentage for the difference between R-dZ_MAX_ and particular PW Doppler derived arrival times.

*Artery*	t_1_-R-dZ_MAX_/t_1_	t_1–2_-R-dZ_MAX_/t_1–2_	t_2_-R-dZ_MAX_/t_2_	t_3_-R-dZ_MAX_/t_3_
*A. carotis dx*.	−32.7 %	−5.1 %	12.6 %	66.5 %
*A. carotis sin*.	−41.1 %	−8.1 %	11.8 %	65.5 %
*A. brachialis dx*.	−22.1 %	3.0 %	19.4 %	47.5 %
*A. brachialis sin*.	−21.5 %	4.3 %	21.0 %	49.6 %
*A. radialis dx*.	−15.5 %	3.4 %	16.9 %	42.0 %
*A. radialis sin*.	−19.4 %	−1.0 %	12.4 %	40.6 %
*A. femoralis dx*.	−21.7 %	5.1 %	21.9 %	47.9 %
*A. femoralis sin*.	−19.1 %	7.7 %	24.4 %	49.5 %
*A. poplitea dx*.	−9.9 %	9.4 %	22.8 %	48.1 %
*A. poplitea sin*.	−16.3 %	4.1 %	18.3 %	45.5 %
*A. tibialis ant. dx*.	−11.2 %	4.3 %	15.8 %	37.1 %
*A. tibialis ant. sin*.	−12.2 %	2.4 %	13.5 %	35.2 %
